# Gastric Outlet Obstruction Caused by a Tapeworm: An Uncommon Presentation of a Common Parasite

**DOI:** 10.4269/ajtmh.20-0661

**Published:** 2020-12

**Authors:** Guda Merdassa Roro, Amir Sultan Seid, David Wong

**Affiliations:** 1 Gastroenterology Division, Department of Internal Medicine, College of Health Sciences, Addis Ababa University, Addis Ababa, Ethiopia;; 2 Department of Gastroenterology and Hepatology, University of Toronto, Toronto, Canada

We present a 38-year-old man from Ethiopia with epigastric pain, repeated vomiting, poor appetite over 1 month, with no history of diarrhea, change in stool color, or fever. He showed no response to omeprazole, and examination was unremarkable, and his stools were negative for parasites and *Helicobacter pylori* antigen. Gastroscopy revealed a bulk of whitish, flat, segmented worm almost completely obstructing the pyloric ring (Figure 1A). There was extensive inflammatory edema involving the antrum and the proximal duodenal mucosa with associated luminal stenosis (B). The worm was gently pushed down into the duodenum using biopsy forceps relieving the obstruction (C). The appearance of the parasite is consistent with a *Taenia* infection. Because the patient had a history of frequently ingesting raw beef and not pork, it was probably *Taenia saginata*. To exclude concomitant pathologies, biopsy specimens were taken from the inflamed areas, the result of which showed chronic lymphocytic gastroduodenitis. The patient was treated with single dose of praziquantel 600 mg and pantoprazole 40 mg twice daily and advised not to eat undercooked meat. On re-evaluation 3 days later, he reported passage of dead worms with stool and improvement of his abdominal pain and vomiting. *Taenia* is a tapeworm acquired by ingesting undercooked beef or pork. Most people with taeniasis are asymptomatic and only become aware of the infection when they pass proglottids in stools. Such gastric obstructive presentations are very uncommon in tapeworm infection, although enteric obstruction with subsequent gastric blockage has been previously reported.^1^ By contrast, bowel obstruction is a well-recognized complication of ascariasis.^2,3^

**Figure 1.**
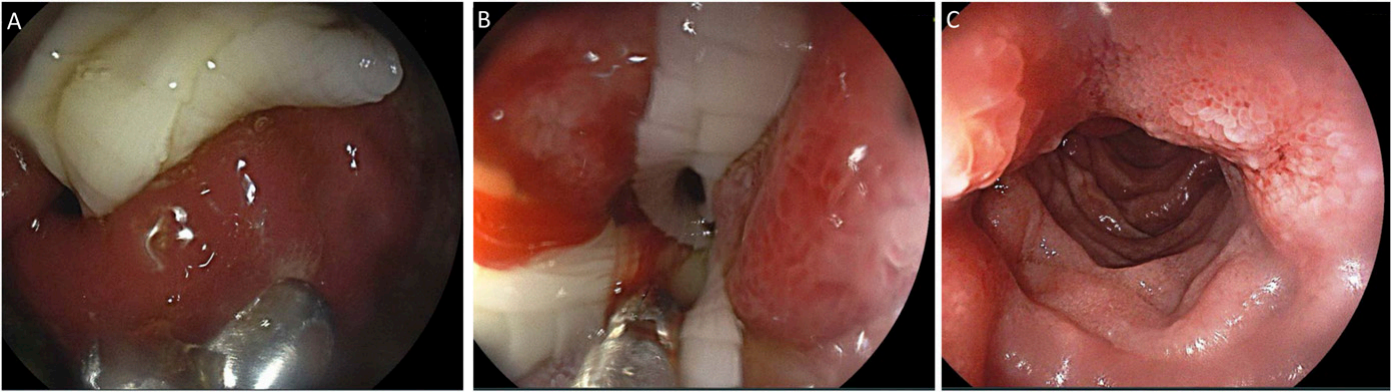
(**A**) Tapeworm nearly completely occluding the pylorus. (**B**) Luminal inflammation with an overlying tapeworm in the duodenum. (**C**) Relief of obstruction after pushing down the tapeworm in the duodenum. This figure appears in color at www.ajtmh.org.

## References

[1] Jesudoss AVS Kaya M Lisa S Rohatgi A, 2012 Wormy surprise! BMJ Case Rep 2012: 2011–2012. 10.1136/bcr.12.2011.5416 PMC3387467 22729345

[2] Uysal E Dokur M, 2017 The helminths causing surgical or endoscopic abdominal intervention: a review article. Iran J Parasitol 12: 156–168. 28761475 PMC5527025

[3] Sotto Mayor J Esperança S, 2015 Gastric *Ascaris* infection. N Engl J Med 373: e18. 26444747 10.1056/NEJMicm1413793

